# Self-Adaptive Trust Based ABR Protocol for MANETs Using *Q*-Learning

**DOI:** 10.1155/2014/452362

**Published:** 2014-08-28

**Authors:** Anitha Vijaya Kumar, Akilandeswari Jeyapal

**Affiliations:** ^1^Dayananda Sagar College of Engineering, Bangalore 560078, India; ^2^Sona College of Technology, Salem 636005, India

## Abstract

Mobile ad hoc networks (MANETs) are a collection of mobile nodes with a dynamic topology. MANETs work under scalable conditions for many applications and pose different security challenges. Due to the nomadic nature of nodes, detecting misbehaviour is a complex problem. Nodes also share routing information among the neighbours in order to find the route to the destination. This requires nodes to trust each other. Thus we can state that trust is a key concept in secure routing mechanisms. A number of cryptographic protection techniques based on trust have been proposed. *Q*-learning is a recently used technique, to achieve adaptive trust in MANETs. In comparison to other machine learning computational intelligence techniques, *Q*-learning achieves optimal results. Our work focuses on computing a score using *Q*-learning to weigh the trust of a particular node over associativity based routing (ABR) protocol. Thus secure and stable route is calculated as a weighted average of the trust value of the nodes in the route and associativity ticks ensure the stability of the route. Simulation results show that *Q*-learning based trust ABR protocol improves packet delivery ratio by 27% and reduces the route selection time by 40% over ABR protocol without trust calculation.

## 1. Introduction

MANETs consist of a group of mobile nodes that communicate over wireless links. Since the nodes are mobile, the network topology may change rapidly and unpredictably over time. A major function in MANETs is the route discovery process, where a route from source node to destination node is discovered in order to transfer data packets. In MANETs, there are three classes of routing protocols: proactive, reactive, and hybrid [1]. The proactive protocols are table driven where each node maintains a route to every other node in the MANETs. Due to limited memory, processing power, and battery capacity, this protocol model is less preferred in MANETs. Reactive routing strategy is popular in wireless ad hoc networks because of its less overhead and on-demand nature. Dynamic source routing (DSR) [2], associativity based routing [3], and ad hoc on-demand distance vector (AODV) are the most popular examples of reactive routing protocols [4]. Hybrid protocols are the combination of the other two and they use the proactive routing procedure to store the route when a routing is initialized and then use the reactive broadcasting to deliver the packet to its destination. Zone routing protocol (ZRP) is the popular hybrid routing protocol [5].

MANETs are deployed in very harsh or abnormal conditions and therefore the probability of network malfunctioning and uncontrolled behavior is very high. They are highly vulnerable to malicious attacks like black hole, grey hole, and Sybil attack [6]. The misbehaving node can be avoided during route discovery process by introducing trust scores of the neighboring nodes through the trust evaluation table. Usage of trust based routing protocol will improve the integrity of the received data at the receiver node [7]. The design of secure and stable routing protocols for MANETs is an active research area. The cooperation among nodes is necessary to sustain the integrity of network operations [8]. However, most of the nodes are selfish or malicious; thus secure routing is vital for protecting the routing protocols against malicious attacks [9]. We use the* Q*-learning technique to enable each node to learn how to adjust its route request forwarding rate according to its trust score. Since* Q*-learning does not always need the detailed model description in computation, it is a widely used reinforcement learning method. It is the best method used to analyze autonomous agents that self-adapt to varying external environments [10, 11]. This paper presents a self-adaptive* Q*-learning based trust ABR (QTABR) that calculates stable and trustworthy nodes for secure routing.

The rest of this paper is organized as follows.  Section 2 gives an overview on the related work. Associativity based routing protocol is described in Section 3.  Section 4 presents trust and* Q*-learning method. Our proposed algorithm is discussed in Section 5.  Section 6 elaborates simulation environment and Section 7 discusses the results on the efficiency of our method. Finally, the last section concludes the paper and gives suggestion for further work in this area.

## 2. Related Work

Reactive routing protocols are more popular due to their dynamic properties. Among reactive routing protocols, associativity based routing (ABR) protocol is preferred because of its link quality. Toh [12] introduced ABR protocol as a loop and deadlock-free protocol with better routes during route reconstruction phase. Choi and Park [13] presented associativity based clustering and query stride protocol where multicast tree discovery, selection, and reconfiguration are based on the association stability. In the work presented by Kim et al.[14], route selection is based on the information obtained by GPS and presence of each mobile node within the transmission range. Their implementation reduces the excessive usage of beacon signals.

Govindan and Mohapatra [15] describe trust computations and trust dynamics such as trust propagation, aggregation, and prediction. Using these trust dynamics [16], various computations are done in distributed and centralized systems. In a multihop wireless network, optimization problems occur in multipath routing [17], link management, relay load, battery power, and secure routing. Hence there is a trade-off between the aforementioned parameters in real time scenarios. To meet these features, many research works are focussed on self-configuration networks based on reinforcement learning [18]. In* Q*-learning based self-configuration (QLS) management and the AODV-Q protocol [19], the QLS management architecture improves packet delivery ratio, end-to-end delay, and other quality of service performance metrics.

Reinforcement technique is also deployed in network management optimization [20–22]. Many works have been done in MANETs using* Q*-learning technique to enhance the performance of packets sent and received. It is mainly based on exploring the environment and learning over time to adapt to network changes. An agent is deployed in the protocol to update the changes in the routing tables.

In [16], reputation for each sensor node is determined using* Q*-learning which helps to find out malicious nodes. The probability of being a malicious node is calculated using statistical and reinforcement techniques. This algorithm detects different types of malicious behaviour in a sensor node.

Reinforcement learning (RL) is a technique used to achieve adaptive routing in MANETs. Temporal difference based RL algorithm is used to achieve higher energy efficiency and less end-to-end delay [23]. In this approach energy-aware route discovery procedure over AODV reactive routing protocol is adapted in which each node adjusts its route request based on its energy table.

## 3. Associativity Based Routing

The ABR protocol is a reactive routing protocol with a metric called the degree of association stability. This associativity is a measure of a node's connectivity relationship with its neighbours over time and space. Each node in the network periodically transmits a beacon to its neighbours signifying its presence. ABR is a uniform routing protocol because of the fact that it provides the same importance to all nodes which participate in routing. A node caches an entry for each neighbour which records the number of beacons received. This information is stored in a variable termed “associativity tick” and is incremented each time a beacon is received. A node is said to exhibit a high state of mobility when it has low associativity ticks with its neighbours. However, if high associativity ticks are observed, the node is in the stable state and this is the ideal point to select the node to perform ad hoc routing. When a node or its neighbour moves to a new location, the node resets the associativity ticks. Associativity threshold [12] is computed as follows:

(1) $$\begin{array}{c} A_{\text{threshold}}=\left(\frac{2r}{pv}\right), \end{array}$$

where *r* is the transmission range, *v* is the migrating speed, and *p* is the beaconing interval.

Association stability results when the number of beacons recorded is greater than *A*_threshold_(*A*_th_). ABR protocol consists of three phases, namely, route discovery, route reconstruction, and route deletion. The first phase consists of broadcast query (BQ) and await-reply (REPLY) cycle. The query packet contains source ID, destination ID, intermediate ID, associativity ticks, hop count, sequence number, and a type field that identifies the type of the message. An intermediate node upon finding it as not the destination, it rebroadcasts the query packet to its neighbours. The destination upon receiving the query packet can find the best route to source by selecting the nodes with high associativity ticks and send the REPLY packet to source. Route reconstruction occurs when a link of an established route changes due to source, destination, and intermediate node migration. In ABR protocol, the selected route is long-lived due to the property of associativity. Even in the case of unexpected movements of nodes, ABR will quickly locate an alternate route. In the last phase, when the source node no longer requires the route to the destination, it sends a route deletion (RD) message and all the intermediate nodes on the way to the destination delete the route from the routing table.

## 4. Trust and* Q*-Learning

Trust has been defined as the belief or confidence or expectation on the honesty, integrity, ability, availability, and quality of service of target node's future activity/behavior [15]. Trust is calculated as a combination of direct and indirect trust [7, 24]. Direct trust is based on the acknowledgements received from the neighbour nodes during the transmission of data packets and control packets. Indirect trust is calculated by the recommendations received from the peer nodes.

**Figure 1:**
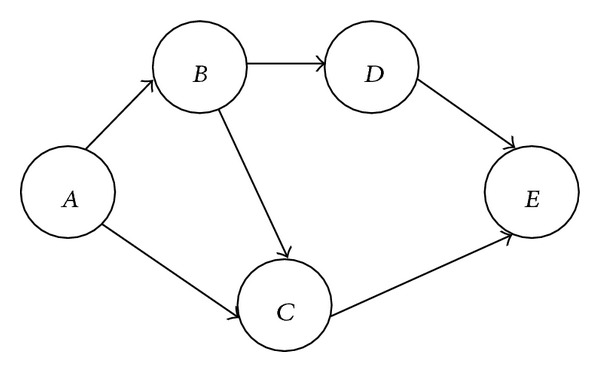
Trust computation.

The following computations of direct and indirect trust values are derived based on the scenario depicted in Figure 1. Direct trust of node *A* with node *B* is computed as follows:

(2) $$\begin{array}{c} T_{\text{DAB}}\left(t\right)=w_{1}\times {\text{CSF}}_{AB}\left(t\right)+w_{2}\times {\text{DSF}}_{AB}\left(t\right). \end{array}$$

In (2) *T*_DAB_(*t*) is the trust of node *B* with respect to the neighbour node *A*, CSF_*AB*_(*t*) is the control signal forwarding ratio, DSF_*AB*_(*t*) is the data signal forwarding ratio between nodes *A* and *B*, and *w*_1_ and *w*_2_ are weights assigned to CSF_*AB*_(*t*) and DSF_*AB*_(*t*), respectively [24]. The weights *w*_1_, *w*_2_ ≥ 0 and *w*_1_ + *w*_2_ = 1.

Indirect trust is computed as follows:

(3) $$\begin{array}{c} T_{\text{IAB}}\left(t\right)=1-\left(1-T_{\text{DAB}}\left(t\right)\right)\;\left(1-T_{\text{ABC}}\left(t\right)\right). \end{array}$$

In (3) *T*_IAB_(*t*) is the indirect trust value of *B* with the recommendation of the neighbour node *C* [25]. *T*_ABC_(*t*) is the trust value sent to *A* by node *C*.

*Q*-learning is a form of model-free reinforcement learning. Usually a RL problem is formulated using Markov decision process (MDP) and is defined as a quintuple (*S*, *A*, *E*, *T*, and *R*), where *S* is the set of states (including finite and infinite states) of the system, *A* is the set of actions that agent performs and affect the system, *E* is the set of external events that the agent has no control over, and *T* is the transition function that associates each state, actions, and events. *T*is  *S* × *A* × *E* × *S* and *R* is the reinforcement or reward function that describes the preference of certain states over other states [15, 22]. It indicates the real value obtained as feedback from environment.

In adaptive routing using reinforcement learning, routing decisions can be changed according to the network conditions [26]. Due to the infrastructureless feature of MANETs, adaptive nature is an advantage to handle the frequent changes in network topology and varying traffic load.

## 5. Proposed* Q*-Learning Model for Trust ABR 

**Figure 2:**
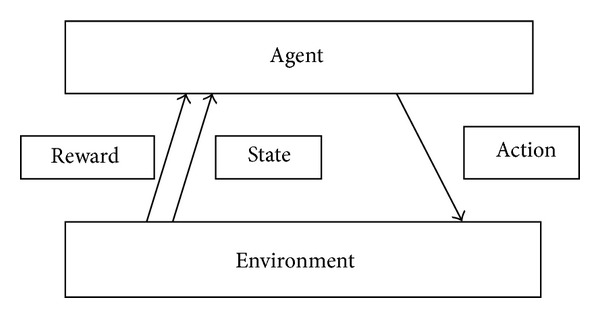
*Q*-learning task.

In* Q*-learning, an action is executed based on the reward received from the environment [19]. In our work, each node is formulated as an agent (Figure 2). It calculates the* Q* value based on the long-term and the aggregated reward. Reward is calculated from the associativity ticks and the trust value of each node with its neighbouring nodes. Generally the* Q*-learning score is defined as

(4) $$\begin{array}{c} Q\left(s,a\right)=\left(1-\propto \right)\cdot Q\left(s,a\right)+\propto \cdot \underset{a}{\max,}Q\left(s_{t+1},a_{t+1}\right), \end{array}$$

where ∝ is the learning rate (0 <  ∝ ≤1) which affects the *Q*-values. In this work the variable *s*_*t*_ represents the present state and *s*_*t*+1_ is the new state. The variable *a*_*t*_ represents the present action; *a*_*t*+1_ represents the action which led to *s*_*t*+1_. In (4), *Q*(*s*, *a*) is the *Q* value derived from the present state-action pair and max⁡_*a*_, ⁡*Q*(*s*_*t*+1_, *a*_*t*+1_) is the maximum *Q* value derived from future state-action pair. In QTABR, each agent has two Q values, namely, *Q*_*p*_ and *Q*_*r*_. The former is the Penalty value of* Q*, when the trust value and associativity ticks of a particular node are less than the threshold. *Q*_*r*_ is the reward value of* Q* when trust value and associativity ticks are more than the threshold. *T*_*v*_ is the trust value calculated by combining the direct and indirect trust value in each agent. A timer is maintained at the source node during the route discovery process to maintain route discovery timeout (RDT).

The values for *Q*_*r*_ and *Q*_*p*_ are computed as follows:

(5) $$\begin{array}{c} Q_{r}=\left(1-\propto \right)\cdot {Q\left(s_{t},a_{t}\right)}_{r}+\propto \cdot \;Q{\left(s_{t+1},a_{t+1}\right)}_{r}, \end{array}$$

where ∝ is given by

(6) $$\begin{array}{c} \propto \;=A_{\text{th}}+T_{v},\\ T_{v}=T_{\text{DAB}}+T_{\text{IAB}}\;\\ Q_{p}=\left(1-\propto \right)\cdot {Q\left(s_{t},a_{t}\right)}_{p}+\propto \cdot \;Q{\left(s_{t+1},a_{t+1}\right)}_{p}. \end{array}$$

Based on the values of *Q*_*r*_ and *Q*_*p*_ each node takes a decision to provide secure and long-lived route. In QTABR routing protocol, when a source node wants to send a message to destination, trust value based on* Q*-learning is computed to establish stable and secure route. It starts the route discovery process by sending a beacon message to its neighbours. Each agent consists of sequence of stages or episodes. Initially the RDT value is set to 30 seconds. The following procedure summarizes the activity of route discovery process.

**Step 1 .**

During the first episode, the source node broadcasts the beacon message.

**Step 2 .**

In the next episode, the neighbouring nodes receive the beacon messages. Association stability is calculated based on the number of beacons recorded which is *A*_th_.

**Step 3 .**

The agent simultaneously calculates the trust threshold value of each neighbouring node *T*_*v*_ as

(7) $$\begin{array}{c} T_{v}=T_{v}+T{}_{\text{NN}}, \end{array}$$

where *T*_NN_ is the trust value of neighbouring nodes. If trust value of a neighbouring node is above threshold value (*T*_*v*_), then the node is accepted as a neighbour. *N*_*a*_ is the total number of accepted neighbouring nodes.

**Step 4 .**

Based on *A*_th_ and *T*_*v*_ value of each node, *Q*_*r*_ and *Q*_*p*_ are calculated in each neighbouring node and broadcast query (BQ) control packet is propagated.

**Step 5 .**

After receiving successful BQ control packet at destination, increase the reward value of that route from the source.

**Step 6 .**

If RDT elapses, increase *Q*_*p*_ value of that route. The agent at the source checks *Q*_*p*_ value against threshold and if the difference is positive, it decides to discard the route. Link delay is automatically eliminated due to the incorporation of RDT.

**Step 7 .**

At the destination node, route selection algorithm (RSA) is used. RSA computes the best route based on metrics such as association stability, hop count, relay load, signal strength, power, and link delay. Trust value is incorporated into the given metrics.

**Step 8 .**

*Q*-learning agent reconfigures the routing parameters like beacon signal intervals, RDT, and RSA.

In the BQ reply control packet of our proposed algorithm, the route quality is added with the trust value as in Figure 4. The aggregated trust value of each node is calculated at the destination. In Table 1, different threshold values for different types of neighbors are shown. If *T*_*v*_ value is less than 0.3, it will be regarded as a malicious node and it would be blacklisted.

**Figure 3:**
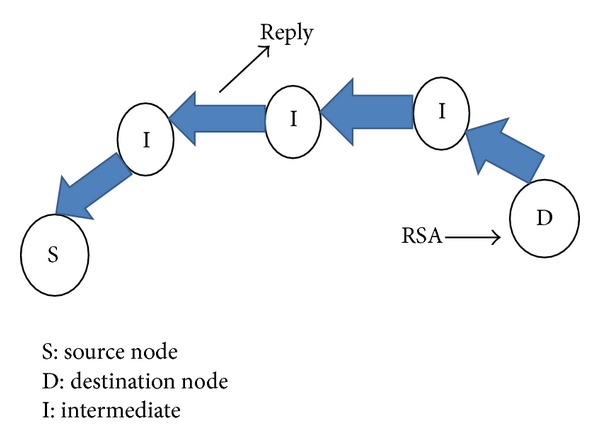
Destination node *D* sends a reply to source.

Figure 3 depicts a destination node sending a reply packet back over the selected route after computing aggregated trust. At the destination, route selection algorithm (RSA) decides the route with higher trust value and associativity threshold.

**Figure 4:**
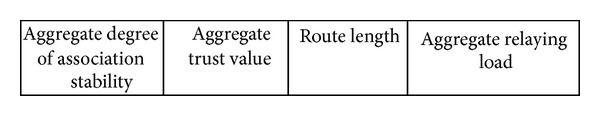
Format of QTABR-BQ reply control packet.

**Figure 5:**
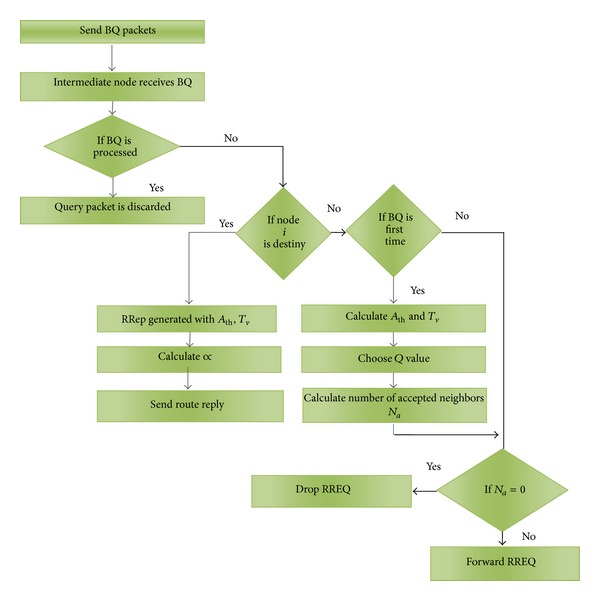
Flow diagram of route discovery phase in QTABR.

Figure 4 depicts the format of broadcast query (BQ) reply control packet in QTABR which includes the aggregated trust value. In BQ reply control packet, route length specifies the length of the route and aggregated relaying load is the total relay load in the specified path. Aggregate degree of association stability is an important parameter, which determines the stability of nodes in the corresponding path.  Figure 5 illustrates the detailed process of route discovery phase in QTABR.

### 5.1. Time Complexity

Our algorithm works in two phases. In phase one associativity ticks and trust value from the beacon signals received from the neighbors using* Q*-learning are computed. The worst case complexity for each node using* Q*-learning for this phase is given by* O *(*e* · *n*), where “*e*” is the number of steps required to reach the secure and stable route and “*n*” is the number of neighboring nodes. During the second phase, each node gets *b*_*i*_ beacon packets. Therefore the time complexity of processing beacon packets is* O *((*e* · *n*) + ∑_*i*=1_^*K*^ *b*_*i*_), where “*K*” is the number of nodes in the path.

## 6. Simulation Environment

We have implemented the proposed* Q*-learning on the top of ABR routing protocol using NS-2 simulator and the simulation summary is given in Table 2. The simulation is carried out in the area of 1000 m∗1000 m with 50 mobile nodes. In each scenario, the nodes move in a random direction using random way point model with a speed randomly chosen within the range of 0–20 m/s. We assumed a presence of 0–40% of malicious nodes in the network. ABR and QTABR results are obtained as the average of 25 runs for each protocol. Maximum pause time is considered for each run. During simulation, QTABR reconfigures RDT between 4 seconds and 12 seconds and beacon messages interval is between 1 second and 12 seconds.

We conduct two sets of experiments to evaluate the performance of our approach. In the first set of experiments, we examined the longevity of the route. In the second set of experiments, malicious node detection and route selection time are examined. The accuracy of malicious detection by trust calculation and overhead of communication in trust computation is considered. Malicious node detection rate and transmission overhead are taken as metrics. Therefore, we distribute malicious nodes randomly and evaluate the performance of our proposed trust based* Q*-learning. In the former study, packet delivery ratio and packet dropping ratio are measured.

### 6.1. Packet Delivery Ratio (PDR)

This ratio gives an indication about network throughput. It is the ratio between the number of packets received (NPR) successfully and number of packets sent (NPS) and as in (8) PDR is directly proportional to long-lived route. Consider

(8) $$\begin{array}{c} \text{PDR}=\frac{\text{NPR}}{\text{NPS}}. \end{array}$$

### 6.2. Packet Dropping Ratio (PD)

This is the percentage of packets dropped during data transmission. It is the ratio between the number of packets dropped (NPD) and number of packets sent (NPS) and as in (9) packet dropping ratio is inversely proportional to longevity of the route. Consider

(9) $$\begin{array}{c} \text{PD}=\frac{\text{NPD}}{\text{NPS}}. \end{array}$$

## 7. Results and Discussions

In the first simulation setup, we measured the packet delivery ratio and packet dropping ratio that directly reflects the longevity of the route. The impact of varying packet size, number of nodes, pause time, and speed on packet delivery ratio is analyzed. For simplicity we mention the algorithm without learning as ABR and the algorithm with learning as QTABR.

### 7.1. Impact of Packet Size on Packet Delivery Ratio

**Figure 6:**
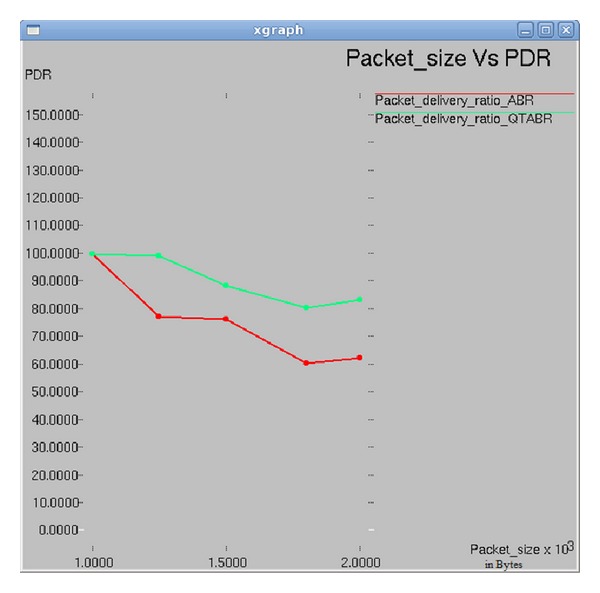
Packet size versus PDR.

In Figure 6, the results are plotted between packet size and packet delivery ratio. The experimental results of ABR show that, as the packet size increases to 1400 bytes, the PDR decreases to 74%. With QTABR, the PDR is 90%, and then it decreases with the increase in packet size. When the packet size increases to 2000 bytes the PDR gradually increases to 85%. Based on the observations, the use of larger packet size can increase the performance of ad hoc networks under QTABR.

### 7.2. Impact of Number of Nodes on Packet Delivery Ratio

**Figure 7:**
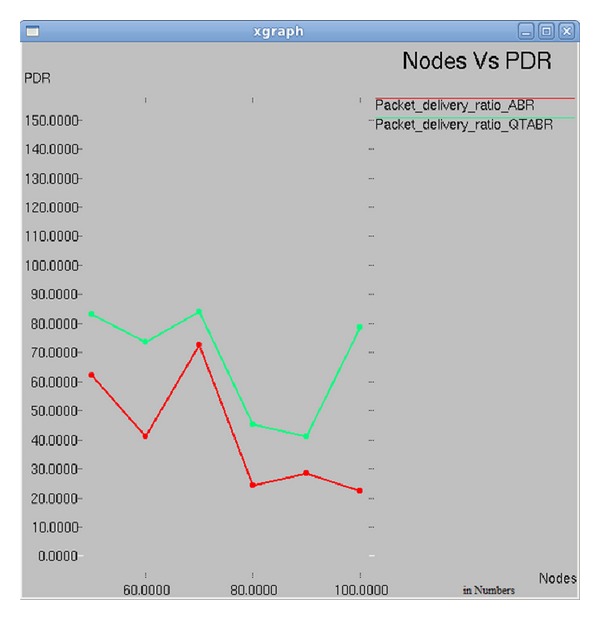
Nodes versus PDR.

Results in Figure 7 show the plot between number of nodes and packet delivery ratio. As the number of nodes increases to 70, PDR value increases steeply to 75% and 85%, using ABR and QTABR, respectively. With the experiments done with ABR, as the number of nodes increases to 80, PDR value drops to 22% and with QTABR, it is 45%. As the number of nodes increases to 100, the PDR value increases to 80% in QTABR's implementation which is 4 times greater than ABR's implementation.

### 7.3. Impact of Pause Time versus PDR

**Figure 8:**
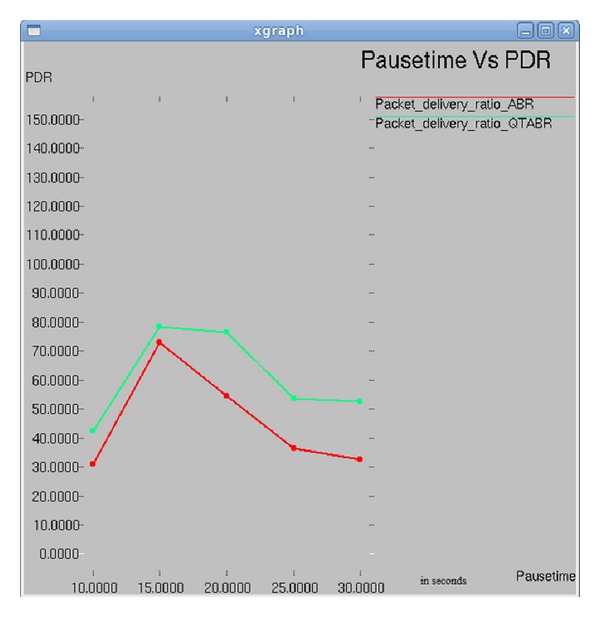
Pause time versus PDR.

Results in Figure 8 depict the plot between pause time and packet delivery ratio. As the pause time increases to 25 ms the PDR drops to 55% and 30% with ABR and QTABR, respectively, but it increases gradually with increase in pause time in QTABR.

### 7.4. Impact of Speed on Packet Delivery Ratio

**Figure 9:**
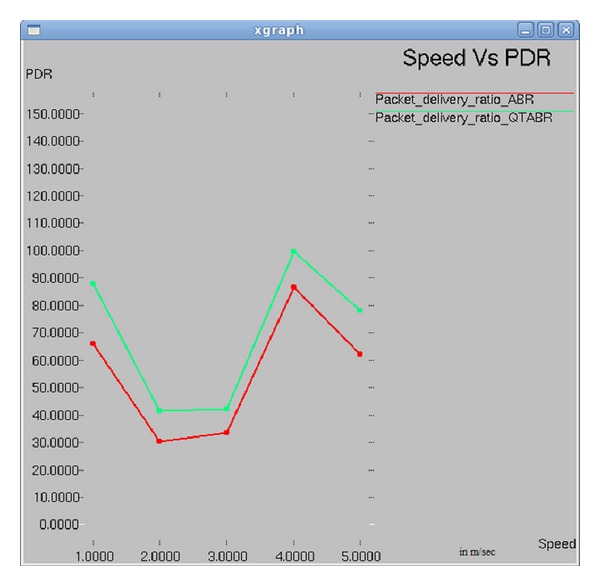
Speed versus PDR.

Figure 9 shows the plot of a graph between the speed time and packet delivery ratio. As the speed increases above 2 m/sec the PDR drops in both of the algorithm implementations. PDR increases to 100% under QTABR when speed is 4 m/sec.

### 7.5. Impact of Packet Size on Packet Dropping Ratio

**Figure 10:**
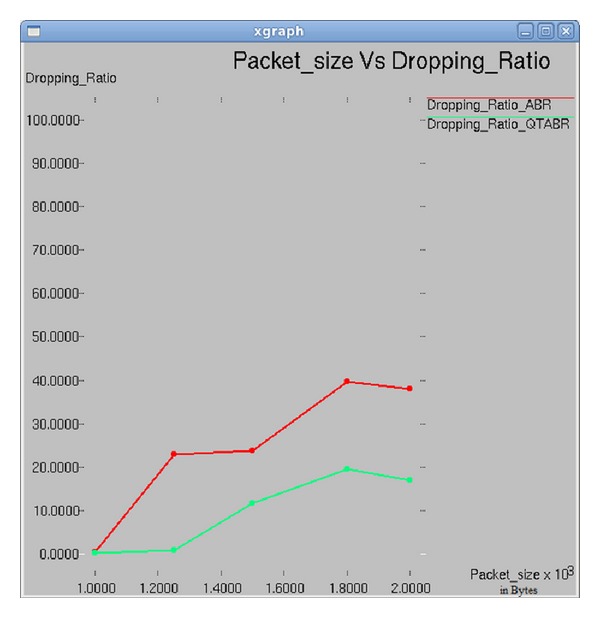
Packet size versus packet dropping ratio.

Figure 10 depicts the graph between packet size and packet dropping ratio. The graph clearly demonstrates the fact of reduced dropping ratio when QTABR is experimented against ABR.

### 7.6. Impact of Number of Nodes on Packet Dropping Ratio

**Figure 11:**
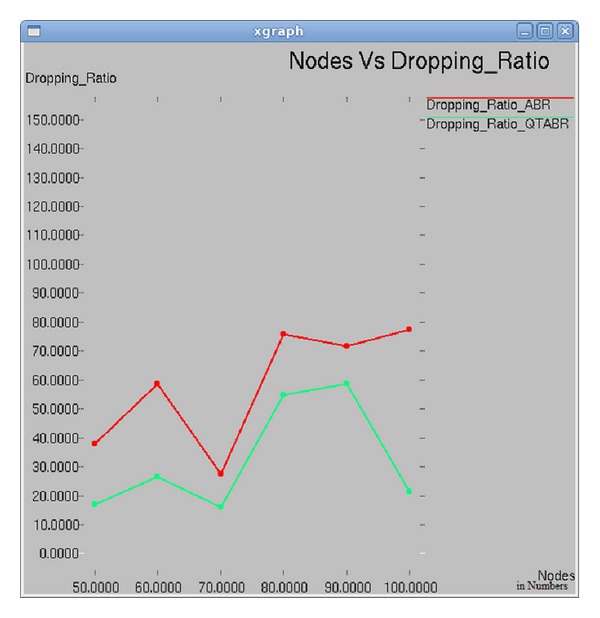
Number of nodes versus packet dropping ratio.

The graph in Figure 11 illustrates the relationship between number of nodes and packet dropping ratio. In both of the algorithms the dropping ratio decreases steeply as the number of nodes reaches 70. When the number of nodes increases to 80, packet dropping ratio is 10% and 25% in ABR and QTABR, respectively. As the number of nodes increases to 100, the packet dropping ratio is 80% under ABR which is 4 times greater than QTABR's implementation.

**Figure 12:**
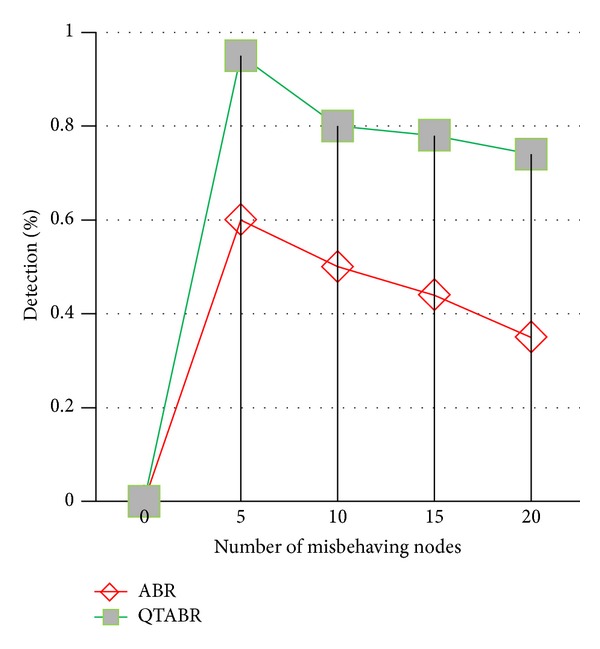
Percentage of detection of malicious nodes.

In the second simulation setup, we increased the number of malicious nodes in the network from five to twenty.  Figure 12 shows the percentage of detected malicious nodes in the network using QTABR. A node will be recognized as malicious node if its trust value (*T*_*v*_) is less than 0.3. When the number of malicious nodes is small, most of the malicious nodes can be recognized using QTABR.

In Figure 12 QTABR routing protocol can explore more malicious nodes and can avoid that route. Thus, selection of secure route is almost 67% to 80% under the proposed condition which is always 10% to 15% more than the existing ABR protocol. Whenever there is a change in route due to link failure, the intermediate nodes should share this information. Rarely, intermediate nodes can also misbehave, and thus the percentage of detection decreases with the increase in the number of malicious nodes. The percentage of detection decreases more in existing ABR as the number of malicious nodes increases.

**Figure 13:**
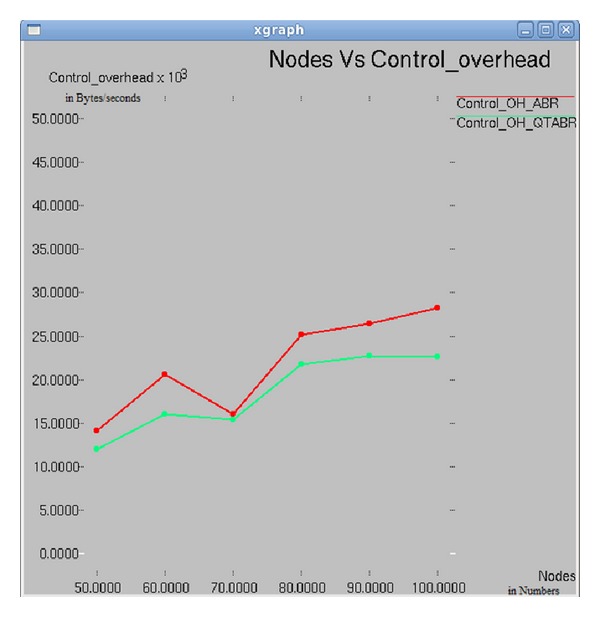
Number of nodes versus control overhead.

In Figure 13, we compared the control overhead of traditional ABR and QTABR. Routing overhead is more, when there is less number of malicious nodes in the network; as the number of malicious nodes increases in a network, the proposed algorithm results in less control overhead. Experimental results suggest that when number of nodes is 70, control overhead is less in both of the routing protocols. It is the optimal value of nodes in the network that produces good output in terms of packet delivery ratio, dropping ratio, and control overhead.

**Figure 14:**
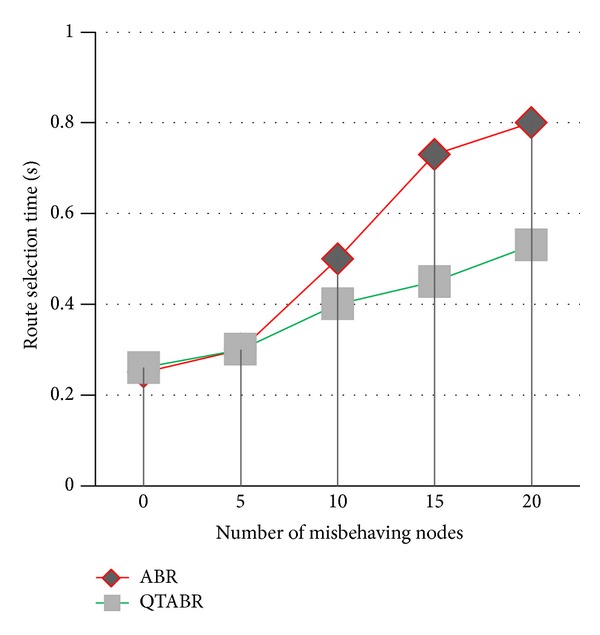
Route selection time.

We have evaluated route selection time, by random distribution of malicious nodes from five to twenty.  Figure 14 shows that as the number of malicious nodes increases the route selection time in the proposed QTABR is less when compared to the existing ABR.

## 8. Conclusion and Future Work

We have proposed a* Q*-learning based trust routing scheme. The proposed scheme is promising as it increases packet delivery ratio and reduces route selection time. We have analyzed the performance of the proposed scheme for various numbers of misbehaving nodes. Security in the routing phase is enhanced by discovering a trustworthy route using QTABR routing protocol. This protocol has proven to offer several advantages. The foremost advantage is the performance of the protocol despite the presence of misbehaving nodes. Secondly, this is applicable to large heterogeneous networks, where the characteristics of the mobile nodes and application demands are different. Thirdly, since the agents are flexible in nature, they can be adapted to any changes with the minimal overhead trade-off. As revealed in this paper, the routing problem in MANETs requires the optimization of many conflicting objectives. This work can be further extended by applying* Q*-learning approach in all stages of ABR to improve the end-to-end routing.

## Figures and Tables

**Table 1:** Trust levels of nodes.

Level	Trust value	Meaning
1	{0,0.29}	Malicious node
2	{0.3,0.59}	Known node
3	{0.6,0.79}	Companion node
4	{0.8,1}	Trustworthy node

**Table 2:** Summary of NS-2 simulation parameters.

Simulation parameters	Values
Simulation area	1000 m ∗ 1000 m
Number of nodes	Mobile nodes (MN) = 100
Malicious nodes	0–20 nodes
Mobility model	Random waypoint
Speed	Uniform (0–20) m/s
Pause time	0,60,120,180,240 in s
Transmission range	350 m
Wireless interface	IEEE 802.11b
Traffic flow	CBR
Transmission power	0.6 W
Reception power	0.3 W
Learning rate ∝	0.6
Simulation duration	10 min (for each run)
